# sleev: An R Package for Semiparametric Likelihood Estimation with Errors in Variables

**DOI:** 10.21105/joss.07320

**Published:** 2025-08-05

**Authors:** Jiangmei Xiong, Sarah C. Lotspeich, Joey B. Sherrill, Gustavo Amorim, Bryan E. Shepherd, Ran Tao

**Affiliations:** 1 Department of Biostatistics, Vanderbilt University Medical Center, USA; 2 Department of Statistical Sciences, Wake Forest University, USA; 3 Brigham Young University, USA; 4 Vanderbilt Genetics Institute, Vanderbilt University Medical Center, USA

## Abstract

Data with measurement error in the outcome, covariates, or both are not uncommon, particularly with the increased use of routinely collected data for biomedical research. With error-prone data, often only a subsample of study data is validated; such settings are known as two-phase studies. The sieve maximum likelihood estimator (SMLE), which combines the error-prone data on all records with the validated data on a subsample, is a highly efficient and robust method to analyze such data. However, given their complexity, a computationally efficient and user-friendly tool is needed to obtain the SMLEs. The R package sleev fills this gap by making semiparametric likelihood-based inference using the SMLEs for error-prone two-phase data in settings with binary and continuous outcomes. Functions from this package can be used to analyze data with error-prone binary or continuous responses and error-prone covariates.

## Statement of Need

Routinely collected data are being used frequently in biomedical research, such as electronic health records. However, these data tend to be error-prone, and using these data without correcting for their error-prone nature could lead to biased estimates and misleading research findings (Duan et al., 2016). To avoid such invalid study results, trained experts carefully verify and extract data elements. However, it is usually only feasible to validate data for a subset of records or variables. After validation, researchers have error-prone, pre-validation data for all records (phase one) and error-free validated data on a subset of records (phase two). Analyses aim to combine the two types of data to obtain estimates that have low bias and are as robust and efficient as possible.

There are several packages for R (R Core Team, 2024) that address measurement error, including augSIMEX (Zhang & Yi, 2019), attenuation (Moss, 2019), decon (Wang & Wang, 2011), eivtools (Lockwood, 2018), GLSME (Hansen & Bartoszek, 2012), mecor (Nab et al., 2021), meerva (Kremers, 2021), mmc (Song, 2015), refitME (Stoklosa et al., 2021), and simex (Lederer & Seibold, 2019). The various R packages reflect many different approaches, such as regression calibration (Wang & Wang, 2011), SIMEX (i.e., simulation-extrapolation) (Lederer & Seibold, 2019), and moment-based corrections (Nab et al., 2021), to mention a few. Nearly all of these existing R packages deal with errors in either the outcome or covariates, but not both, and none of these packages permits efficient inference that incorporates both the error-prone phase-one data and the validated phase-two data.

The sieve maximum likelihood estimator (SMLE) is an estimator that analyzes two-phase data by combining the error-prone data on all records with the validated data on a subsample. By leveraging all available data, the SMLE operates with high efficiency (Lotspeich et al., 2022; Tao et al., 2021). Since it does not make any parametric assumptions on the error model, the SMLE is also robust. For example, Tao et al. (2021) performed a set of simulations highlighting the SMLE’s robustness to different error mechanisms including settings where the errors had non-zero mean or were multiplicative. Moreover, the SMLE allows error-prone outcome and error-prone covariates in the same model. Still, in practice these estimators can be difficult to implement, as they involve approximating nuisance conditional densities using B-splines (Schumaker, 2007) and then maximizing the semiparametric likelihood via a sophisticated EM algorithm (Tao et al., 2017). Here, we present the R package sleev, which makes the SMLE readily applicable for practitioners in a user-friendly way. sleev integrates and extends primitive R packages, logreg2ph and TwoPhaseReg, developed with the original methods papers (Lotspeich et al., 2022; Tao et al., 2021). These two packages lacked proper documentation and were difficult to use. logreg2ph was also computationally slow.

To promote the use of the SMLE, extensive work has been done to create sleev, a computationally efficient and user-friendly R package to analyze two-phase, error-prone data. Specifically, in sleev we rewrote the core algorithms of logreg2ph in C++ to speed up the computation, and we unified the syntax across functions. To compare the computational times, we set up simulations with the same code in the package vignette. The simulations included phase-one and phase-two sample sizes of 2087 and 835, respectively, and were performed on a 64-bit Linux OS machine with 8G memory. Across 100 simulations, the previous logreg2ph took an average of 289.44 seconds with a standard deviation of 8.83 seconds to perform the analysis, while the corresponding new function in sleev only took an average of 122.32 seconds with a standard deviation of 8.18 seconds.

## SMLE for Linear Regression

In this section, we briefly introduce the SMLE for linear regression. Suppose that we want to fit a standard linear regression model for a continuous outcome Y and covariates $X:Y=\alpha +\beta^{\text{T}}X+\epsilon ,$, where $\epsilon \sim N\left(0,\sigma^{2}\right)$. Our goal is to obtain estimates of $\theta ={\left(\alpha ,\beta^{\text{T}},\sigma^{2}\right)}^{\text{T}}$. When we have error-prone data, Y and ***X*** are unobserved except for a subset of validated records. For unvalidated records (the majority), only the error-prone outcome $Y^{*}=Y+W$ and covariates $X^{*}=X+U$ are observed in place of Y and ***X***, where W and ***U*** are the errors for the outcome and covariates, respectively. We assume that W and ***U*** are independent of ϵ. With potential errors in our data, a naive regression analysis using error-prone variables Y* and X* could render misleading results (Fuller, 2009).

We assume that the joint density of the complete data $\left(Y^{*},X^{*},W,U\right)$ takes the form

$$\begin{array}{l} P\left(Y^{*},X^{*},W,U\right)=P\left(Y^{*}|X^{*},W,U\right)P\left(W,U|X^{*}\right)P\left(X^{*}\right)\\ \;\;\;\;\;=P_{\theta }(Y|X)P\left(W,U|X^{*}\right)P\left(X^{*}\right), \end{array}$$

where $P\left(\cdot \right)$ and $P\left(\cdot \left|\cdot \right.\right)$ denote density and conditional density functions, respectively. Specifically, $P_{\theta }(Y|X)$ then refers to the conditional density function of the linear regression model of Y given ***X***. Denote the validation indicator variable by *V*, with *V* = 1 indicating that a record was validated and *V* = 0 otherwise. For records with *V* = 0, their measurement errors (W, U) are missing, and therefore their contributions to the log-likelihood can be obtained by integrating out W and ***U***.

Let $\left(Y_{i}^{*},X_{i}^{*},W_{i},U_{i},V_{i},Y_{i},X_{i}\right)$ for i=1,…,n denote independent and identically distributed realizations of $\left(Y^{*},X^{*},W,U,V,Y,X\right)$ in a sample of *n* subjects. Then, the observed-data log-likelihood is proportional to

(1) $$\begin{array}{l} \sum_{i=1}^{n}V_{i}\left\{\log P_{\theta }\left(Y_{i}|X_{i}\right)+\log P\left(W_{i},U_{i}|X_{i}^{*}\right)\right\}\\ +\sum_{i=1}^{n}\left(1-V_{i}\right)\log\left\{\iint P_{\theta }\left(Y_{i}^{*}-w|X_{i}^{*}-u\right)P\left(w,u|X_{i}^{*}\right)dwdu\right\}, \end{array}$$

where $P\left(X^{*}\right)$ is left out, because the error-prone covariates are fully observed and thus $P\left(X^{*}\right)$ can simply be estimated empirically. We estimate the unknown measurement error model, $P\left(W_{i},U_{i}|X_{i}^{*}\right)$, using B-spline sieves. Specifically, we approximate $P\left(w,u|X_{i}^{*}\right)$ and log $P\left(W_{i},U_{i}|X_{i}^{*}\right)$ by $\sum_{k=1}^{m}\text{I}\left(w=w_{k},u=u_{k}\right)\sum_{j=1}^{s_{n}}B_{j}^{q}\left(X_{i}^{*}\right)p_{kj}$ and $\sum_{k=1}^{m}\text{I}\left(W_{i}=w_{k},U_{i}=u_{k}\right)\sum_{j=1}^{s_{n}}B_{j}^{q}\left(X_{i}^{*}\right)\log p_{kj}$, respectively. Here, $\left\{\left(w_{1},u_{1}\right),\ldots ,\right.\left.\left(w_{m},u_{m}\right)\right\}$ are the *m* distinct observed (*W*, ***U***) values from the validation study, $B_{j}^{q}\left(X_{i}^{*}\right)$ is the jth B-spline basis function of order *q* evaluated at $X_{i}^{*}$, *s*_*n*_ is the dimension of the B-spline basis, and *p_kj_* is the coefficient associated with $B_{j}^{q}\left(X_{i}^{*}\right)$ and $\left(w_{k},u_{k}\right)$. The expression (1) is now approximated by

(2) $$\begin{array}{l} \sum_{i=1}^{n}V_{i}\left[\log P_{\theta }\left(Y_{i}|X_{i}\right)+\sum_{k=1}^{m}\left\{\text{I}\left(W_{i}=w_{k},U_{i}=u_{k}\right)\sum_{j=1}^{s_{n}}B_{j}^{q}\left(X_{i}^{*}\right)\log p_{kj}\right\}\right]\\ +\sum_{i=1}^{n}\log\left[\sum_{k=1}^{m}\left\{P_{\theta }\left(Y_{i}^{*}-w_{k}|X_{i}^{*}-u_{k}\right)\sum_{j=1}^{s_{n}}B_{j}^{q}\left(X_{i}^{*}\right)\log p_{kj}\right\}\right]. \end{array}$$

The maximization of expression (2) is carried out through an EM algorithm to find the SMLEs $\hat{\theta }$ and ${\hat{p}}_{kj}$. The covariance matrix of the SMLE $\hat{\theta }$ is obtained through the method of profile likelihood (Murphy & Van der Vaart, 2000).

The SMLEs for logistic regression are similar to linear regression and described in the package vignette, and the theoretical properties can be found in Lotspeich et al. (2022).

## Functionalities of the sleev R Package

The sleev package provides a user-friendly way to obtain the SMLEs and their standard errors. The package can be installed from CRAN or GitHub. The sleev package includes two main functions: linear2ph() and logistic2ph(), to fit linear and logistic regressions, respectively, under two-phase sampling with an error-prone outcome and covariates. The input arguments are similar for the two functions and listed in Table 1. In addition to the arguments for error-prone and error-free outcome and covariates, the user needs to specify the B-spline matrix $B_{j}^{q}\left(X_{i}^{*}\right)$ to be used in the estimation of the error densities.

## Example: Case study with mock data

For demonstration, the sleev package includes a dataset constructed to mimic data from the Vanderbilt Comprehensive Care Clinic (VCCC) patient records from Giganti et al. (2020). Table 2 describes the variables in this dataset.

We now illustrate how to obtain the SMLEs using the sleev package with the mock.vccc dataset. Specifically, we show how to fit a linear regression model in the presence of errors in both the outcome and covariates using the linear2ph() function. Situations with more covariates and examples with logistic regression are included in the package vignette.

This example fits a linear regression model with CD4 count at antiretroviral therapy (ART) initiation regressed on viral load (VL) at ART initiation, adjusting for sex at birth. Both CD4 and VL are error-prone, partially validated variables, whereas sex is error-free. Because of skewness, we often transform both CD4 and VL. In our analysis, CD4 was divided by 10 and square root transformed, and VL was log_10_ transformed:


library(“sleev”)
data(“mock.vccc”)
mock.vccc$CD4_val_sq10 <- sqrt(mock.vccc$CD4_val / 10)
mock.vccc$CD4_unval_sq10 <- sqrt(mock.vccc$CD4_unval / 10)
mock.vccc$VL_val_l10 <- log10(mock.vccc$VL_val)
mock.vccc$VL_unval_l10 <- log10(mock.vccc$VL_unval)


To obtain the SMLEs, we first need to set up the B-spline basis for the error-prone covariate VL_unval_l10 (the transformed VL variable from phase one) and Sex. The spline2ph() function in the sleev package can set up the B-spline basis, and combine it with the input data for the final analysis. Here, we use a cubic B-spline basis with the degree = 3 argument. The size of the basis *s_n_* is set to be 20, specified through the size = 20 argument. More details regarding order and size selection, as well as run time comparison of B-spline basis, are discussed in the package vignette. To allow possible heterogeneity in error distribution between males and females, we can set up B-spline basis separately and proportionally for the two Sex groups by specifying argument group = “Sex”. The described B-spline basis is constructed as follows.


sn <- 20
data.linear <- spline2ph(x = “VL_unval_l10”, data = mock.vccc, size = sn,
				 degree = 3, group = “Sex”)


Alternatively, if the investigator has prior knowledge that the errors in VL_unval_l10 are likely to be homogeneous, one may fit a simpler model by not stratifying the B-spline basis by Sex.

Having constructed the B-spline basis, the SMLEs can be obtained by running the linear2ph() function on data. linear, as shown in the code below. Again, the inputs are explained in Table 1. The fitted SMLEs are stored in a list object of class linear2ph. Here, we assign the fitted SMLEs to the variable name res_linear. The list of class linear2ph contains five components: coefficient, covariance, sigma, converge, and converge_cov.


res_linear <- linear2ph(y_unval = “CD4_unval_sq10”, y = “CD4_val_sq10”,
		x_unval = “VL_unval_l10”, x = “VL_val_l10”,
		z = “Sex”, data = data. linear,
		hn_scale = 1, se = TRUE, tol = 1e-04,
		max_iter = 1000, verbose = FALSE)


We should first check if the EM algorithms for estimating the regression coefficients and their covariance matrix converged by using the print() for class linear2ph directly.


> res_linear
Call:
linear2ph(y_unval = “CD4_unval_sq10”, y = “CD4_val_sq10”,
	x_unval = “VL_unval_l10”, x = “VL_val_l10”, z = “Sex”,
	data = data. linear, hn_scale = 1, se = TRUE,
	tol = 1e-04, max_iter = 1000, verbose = FALSE)
The parameter estimation has converged.
Coefficients:
 Intercept VL_val_l10 Sex
 4.8209166 −0.1413168 0.2727984


The summary() function for the object of class linear2ph returns the estimated coefficients, their standard errors, test statistics, and *p*-values as follows:


> summary(res_linear)
Call:
linear2ph(y_unval = “CD4_unval_sq10”, y = “CD4_val_sq10”,
	x_unval = “VL_unval_l10”, x = “VL_val_l10”, z = “Sex”,
	data = data. linear, hn_scale = 1, se = TRUE,
	tol = 1e-04, max_iter = 1000, verbose = FALSE)
Coefficients:
	     Estimate   SE Statistic         p-value
Intercept   4.8209166 0.15865204 30.386729 0.0000000000
VL_val_l10 −0.1413168 0.03983406 −3.547636 0.0003887047
Sex        0.2727984 0.10888178	2.505455 0.0122294098


## Figures and Tables

**Table 1:** Main arguments for the linear2ph() and logistic2ph() functions

Argument	Description
y_unval	Column name of unvalidated outcome in the input dataset.
Y	Column name of validated outcome in the input dataset. NAs in the input will be counted as individuals not selected in phase two.
x_unval	Column names of unvalidated covariates in the input dataset.
x	Column names of validated covariates in the input dataset. NAs in the input will be counted as individuals not selected in phase two.
z	Column names of error-free covariates in the input dataset.
data	Dataset generated from sleev::spline2ph().
hn_scale	Scale of the perturbation constant in the variance estimation via the method of profile likelihood. The default is 1.
se	Standard errors of the parameter estimators will be estimated when set to TRUE. The default is TRUE.
tol	Convergence criterion. The default is 0.0001.
max_iter	Maximum number of iterations in the EM algorithm. The default is 1000.
verbose	Print analysis details when set to TRUE. The default is FALSE.

**Table 2:** Data dictionary for mock.vccc

Name	Status	Type	Description
ID	error-free		Patient ID
VL_unval	error-prone	continuous	Viral load (VL) at antiretroviral therapy (ART)
VL_val	validated	continuous	initiation
ADE_unval	error-prone	binary	Had an AIDS-defining event (ADE) within one
ADE_val	validated	binary	year of ART initiation: 1 - yes, 0 – no
CD4_unval	error-prone	continuous	CD4 count at ART initiation
CD4_val	validated	continuous	
Prior_ART	error-free	binary	Experienced ART before enrollment: 1 - yes, 0 - no
Sex	error-free	binary	Sex at birth of patient: 1 - male, 0 - female
Age	error-free	continuous	Age of patient
